# Primed to Sleep: The Dynamics of Synaptic Plasticity Across Brain States

**DOI:** 10.3389/fnsys.2019.00002

**Published:** 2019-02-01

**Authors:** Julie Seibt, Marcos G. Frank

**Affiliations:** ^1^Surrey Sleep Research Centre, University of Surrey, Guildford, United Kingdom; ^2^Department of Biomedical Sciences, Elson S. Floyd College of Medicine, Washington State University Spokane, Spokane, WA, United States

**Keywords:** sleep, experience, plasticity, tagging, excitability, priming, consolidation

## Abstract

It is commonly accepted that brain plasticity occurs in wakefulness *and* sleep. However, how these different brain states work in concert to create long-lasting changes in brain circuitry is unclear. Considering that wakefulness and sleep are profoundly different brain states on multiple levels (e.g., cellular, molecular and network activation), it is unlikely that they operate exactly the same way. Rather it is probable that they engage different, but coordinated, mechanisms. In this article we discuss how plasticity may be divided across the sleep–wake cycle, and how synaptic changes in each brain state are linked. Our working model proposes that waking experience triggers short-lived synaptic events that are necessary for transient plastic changes and mark (i.e., ‘prime’) circuits and synapses for further processing in sleep. During sleep, synaptic protein synthesis at primed synapses leads to structural changes necessary for long-term information storage.

## Introduction

Experience-dependent plasticity, including memory, is divided temporally into an induction (encoding) and consolidation phase. A general consensus in the field is that while encoding of new information occurs during wakefulness, sleep integrates and stabilizes the resulting plastic changes (i.e., consolidation) (Frank and Benington, 2006; Rasch and Born, 2013). This would be adaptive because experience would not trigger immediate consolidation of all incoming information, and thus avoid simultaneous and complex regulation of different cascades of information storage (Redondo and Morris, 2011). New information processing and storage in the brain are highly complex and dynamic processes that involve changes at different levels, from single synapses and molecules to entire circuits. At the single neuron level, this translates into waves of post-translational protein modifications and new gene expression that ultimately promote long-term stabilization of changes in synaptic strength (Flavell and Greenberg, 2008). These processes likely involve different but interdependent mechanisms that are divided across wakefulness and sleep. They are likely to be different because wake and sleep are fundamentally different brain states. They should also be interdependent to ensure that information is detected, encoded and consolidated across brain states.

While existing theories on sleep function posit different roles for wake and sleep in experience-dependent plasticity, they do not systematically distinguish between mechanisms known to participate in plasticity induction and consolidation. For example, synaptic potentiation, which has been proposed to occur during either wake (Tononi and Cirelli, 2014) or sleep (Rasch and Born, 2013), is a dynamic process that is initially labile and relies on specific molecular mechanisms for its long lasting expression. There is also an abundant literature showing that both wake and sleep promote molecular mechanisms of synaptic plasticity (for recent reviews, see da Costa Souza and Ribeiro, 2015; Puentes-Mestril and Aton, 2017; Almeida-Filho et al., 2018; Navarro-Lobato and Genzel, 2018). What is less clear is how the mechanisms of plasticity induction and consolidation are divided across brain states. Therefore, it is important to delineate the plastic processes that occur in different brain states and to determine how changes in one brain state link to and influence changes in another.

In this paper, we discuss new ideas about how plasticity mechanisms are divided across the sleep–wake cycle and how plastic events in one brain state set in motion plastic changes in another. More specifically we propose that waking experience promotes two parallel events that recruit shared mechanisms: transient plastic changes and the priming of neurons for further modification in sleep. Priming refers to experience-dependent mechanisms that mark neurons in a synapse- and circuit-specific manner for further modifications. During sleep, reactivation of primed circuits leads to more permanent changes in synaptic weights via stabilization of structural plasticity. Structural plasticity stabilization involves a set of localized molecular mechanisms that transforms the transient priming instructions into lasting morphological changes at synapses. We conclude with predictions, open questions and future directions.

## Plasticity Induction During Wakefulness: Labile Changes and Neuronal Priming

When considering experience-dependent plasticity, it is important to understand the nature and timeline of processes that underlie plasticity induction and stabilization. New experience activates a cascade of events in neurons whereby each step triggers specific molecular mechanisms necessary for the full expression of long-term synaptic plasticity. The lasting nature of plasticity-related changes in neurons is traditionally viewed to depend on activation of new gene transcription and translation (Flavell and Greenberg, 2008; Hernandez and Abel, 2008). Several molecular and anatomical studies show that waking leads to labile synaptic changes associated with the induction process. Some of these same events may also prime neurons for further modification in sleep. We discuss these ideas in more detail in the following sections.

### Transient Plastic Changes During Wakefulness

Findings *in vivo* and *in vitro* indicate that the plasticity induction process triggers short-lived changes that mainly depend on post-translational modifications of existing proteins and rapid morphological alterations of dendritic spines (Lang et al., 2004; Hernandez and Abel, 2008; Bailey et al., 2015). At the molecular level, many forms of plasticity induction rely on *N*-methyl-D-aspartate receptors (NMDARs) activation leading to intracellular calcium (Ca^2+^) influx and rapid activation of various protein kinases [e.g., calcium/calmodulin dependent kinases (CaMKs), protein kinases A (PKA), and C (PKC), extracellular signal-regulated protein kinase (ERK)] and phosphatases [e.g., protein phosphatase 1 (PP1), calcineurin] (Ho et al., 2011). Enzymatic activation triggers two main modifications at synapses: changes in α-amino-3-hydroxy-5-methyl-4-isoxazolepropionic acid receptors (AMPARs) number and activity (Hayashi et al., 2000; Esteban et al., 2003; Yang et al., 2008) and morphological changes of spines. Experience and long-term potentiation (LTP) protocols are associated with rapid (within 1–2 h) changes in spine volume and number. These changes are transient and only a fraction of newly formed spines will persist over time (Xu et al., 2009; Yang et al., 2009). This is consistent with the idea that stabilization of structural plasticity induced by experience can take several hours to weeks, and involves both strengthening and weakening of selected synapses (Hofer et al., 2009; Xu et al., 2009; Yang et al., 2009; Sanders et al., 2012; El-Boustani et al., 2018). This initial modification of spine structure is thought to rely mainly on phosphorylation-dependent changes in actin cytoskeleton dynamics rather than new protein synthesis (Lang et al., 2004; Bhatt et al., 2009; Okamoto et al., 2009; Caroni et al., 2014; Bailey et al., 2015).

In addition to these protein synthesis-independent synaptic changes, experience also rapidly induces new gene expression which is the first step toward consolidation of plastic changes in neurons (Flavell and Greenberg, 2008). In particular, gene transcription activation occurs within minutes in specific neuronal ensembles activated by experience (Minatohara et al., 2016; Lisman et al., 2018). The two main signatures of experience-dependent transcription are the phosphorylation of cAMP response element binding protein (CREB) and the transient expression of Immediate Early Genes (IEGs) (Flavell and Greenberg, 2008; Caroni et al., 2014; Minatohara et al., 2016). Collectively, those data suggest that at the molecular and structural levels, early forms of plasticity are initially fragile, require covalent change of pre-existing proteins and rapid IEG transcription, but are largely independent of translation.

Studies in animals and humans show that many forms of memory are labile and susceptible to interference *until sleep occurs*, after which they are resistant to further disruption (reviewed in Aton et al., 2009b). A very recent study by Wang et al. (2018) demonstrated that, compared to sleep, wakefulness globally increases phosphorylation in the brain. Interestingly, among the proteins that showed increased phosphorylation, most of them were synaptic proteins associated with pre-synaptic short-term plasticity (e.g., Syn1, Rims1), post-synaptic density structure (e.g., scaffolding proteins, actin remodeling), glutamate receptors (e.g., Grin2b/5), or inhibition of translation (e.g., AMPK) (Wang et al., 2018). *In vitro* measures show that extended wakefulness (i.e., sleep deprivation, SD) impairs hippocampal LTP dependent on cAMP/PKA/CREB signaling, but not LTP that requires new translation (Vecsey et al., 2009). In the developing visual cortex, plasticity *in vivo* induced during waking is independent of translation, as inhibition of kinases critical for translation activation (i.e., mTOR, ERK, MNK) has no effect on waking plasticity (Seibt et al., 2012; Dumoulin et al., 2015). Finally, the stabilization of learning-induced spine formation and pruning in the rodent cortex does not occur during wakefulness, and is instead only detectable after sleep (Yang et al., 2014; Li et al., 2017). Therefore, plasticity during wakefulness is similar to ‘early’ forms of plasticity *in vitro* that are also labile and translation independent. In the next section, we will describe how these early molecular changes outline physiological processes involved in cell-wide and synapses-specific priming in neurons and circuits activated by experience.

### Bridging Brain States Through Priming Mechanisms During Wakefulness

**FIGURE 1:**
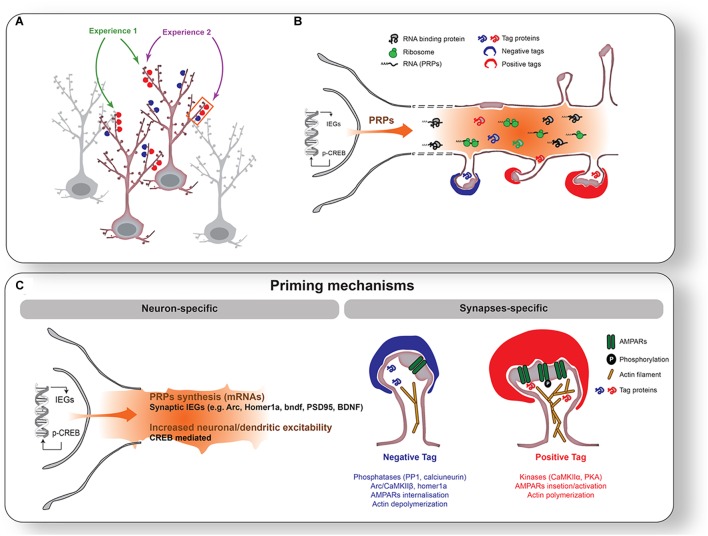
Priming mechanisms during wakefulness. **(A)** Individual neurons and synapses activated by two different experiences (1 [green] and 2 [purple]). Experience 1 activates synapses onto two different neurons, while Experience 2 activates different synapses on the same neuron. In this example, Experiences 1 and 2 will stimulate one common neuron and a subset of common synapses on the same dendritic branch. Experience will increase excitability in selected neurons (highlighted with red outline) and tag synapses positively (red cap on spines) or negatively (blue cap on spines). **(B)** Enlargement of the segment of dendrites outlined with an orange rectangle in **(A)**. CREB activation and IEGs expression will be triggered in activated neurons. This leads to a transcription of Plasticity Related Products (PRPs: e.g., arc, bdnf, PSD-95, Homer1a) transported to dendrites. **(C)** Illustration of mechanisms of neuronal (*left*) and synaptic (*right*) priming. Neuronal priming is mainly supported by CREB-dependent increase in neuronal/dendritic excitability. Synaptic priming is achieved by tagging mechanisms such as post-translational modifications of receptors, enzymes, and actin filaments.

If wakefulness and sleep are partners in synaptic plasticity, how does the induction of plasticity during wakefulness lead to secondary modifications during sleep? The answer may lie in a phenomenon known as ‘metaplasticity’ (Hulme et al., 2013; Yee et al., 2017). Metaplasticity refers to neural changes that influence the capacity for subsequent synaptic plasticity (“*plasticity of synaptic plasticity*” (Abraham and Bear, 1996)). Among the various forms of metaplasticity, synaptic tagging (Frey and Frey, 2008; Redondo and Morris, 2011) and changes in neuronal excitability (Benito and Barco, 2010) provide two types of mechanisms that mark or *prime* synapses and neurons engaged in the learned experience, respectively. Importantly, neuronal circuits are primed according to their activation history (i.e., weak or strong) and thus allow delayed consolidation while maintaining cell- and input-specificity, as well as bidirectionality of plastic changes. Although current metaplasticity mechanisms do not consider sleep and wakefulness and have been mainly integrated into models of memory allocation (Rogerson et al., 2014; Lisman et al., 2018), it is easy to see how a similar process could provide a molecular bridge between plastic changes in one brain state and those in another (Figure 1).

#### Synaptic Tagging

Long-term synaptic plasticity does not appear to involve a change in the total number of synapses but is instead accompanied by the formation and loss of synapses (Popov et al., 2004; Bourne and Harris, 2011; El-Boustani et al., 2018). This is consistent with the observation that both spine formation and pruning occur across wake and sleep (Maret et al., 2011; Yang and Gan, 2012; Li et al., 2017). But how does the sleeping brain remember and select synapses for strengthening or for pruning? Here the Synaptic Tagging and Capture hypothesis (STC) (Frey and Morris, 1997; Redondo and Morris, 2011) provides an attractive framework. The STC proposes that learning creates the potential for long-term synaptic changes by setting ‘tags’ at remodeling synapses. Later reactivation (within hours) of the neuronal network surrounding tagged synapses promotes the capture and translation of Plasticity Related Products (PRPs) leading to a final stabilization of synaptic weight change while maintaining input-specificity (Redondo and Morris, 2011). Since tags can be positive or negative, this marks synapses for further spinogenesis/strengthening or pruning/weakening upon reactivation.

##### Tags

While their identity is still under investigation, tags are thought to be localized molecular processes that render synapses temporally permissive for PRPs capture (Redondo and Morris, 2011). Tags can include transient protein modifications that occur at synapses (e.g., phosphorylation/dephosphorylation). Pharmacology and genetic manipulations have shown that CaMKIIα may act as a positive tag necessary for later LTP/spine enlargement (Okamoto et al., 2009; Redondo et al., 2010; Redondo and Morris, 2011). Due to its ability to autophosphorylate, CaMKIIα is a strong tag candidate as its activation can last for several hours and span across different brain states. Furthermore, CaMKIIα autophosphorylation is detectable during wake (Vyazovskiy et al., 2008) and early sleep following experience-dependent plasticity induction in the cortex (Aton et al., 2009a) and hippocampus (Blanco et al., 2015). The kinase PKA may also act as a positive tag (Young et al., 2006; Moncada et al., 2011). PKA is rapidly activated upon plasticity induction and also appears to remain active for several hours following experience (Aton et al., 2009a) or learning (Abel et al., 1997; Bourtchouladze et al., 1998). Consistent with this role, PKA inhibition during sleep only affects the potentiation, but not the depression, of neuronal activity normally seen in a physiological model of developmental plasticity in the visual cortex (i.e., ocular dominance plasticity [ODP]) (Aton et al., 2009a). Negative (or ‘inverse’) tags will mark synapses for synaptic weakening and include activation of phosphatases and kinase isoforms. The phosphatase calcineurin, for example, may act as inverse tag for long-term depression (LTD)/spine shrinkage induction (Zhou et al., 2004; Redondo and Morris, 2011). The CaMKII isoform CaMKIIβ has also been proposed to act as inverse tag, as it can interact with negative PRPs such as Arc (Okuno et al., 2012). The function of a tag can also vary depending on its action on actin filaments. It has therefore been recently proposed that a core tagging mechanism are changes in actin dynamics (Okamoto et al., 2009; Redondo and Morris, 2011; Szabó et al., 2016).

##### PRPs

**FIGURE 2:**
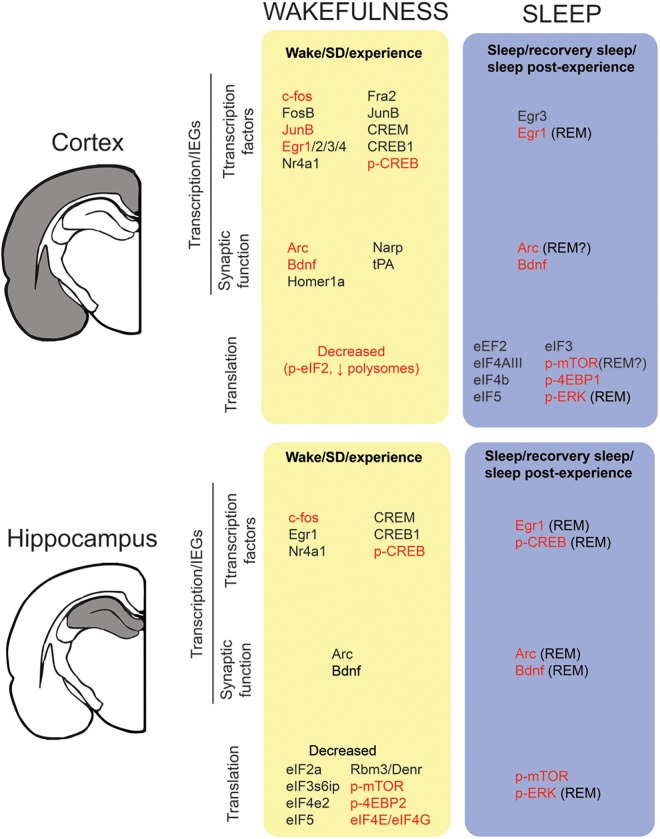
Transcription and translation across sleep and wake in the cortex and hippocampus. We report the state-dependent regulation of (1) immediate early genes (IEGs) from the transcription factors and synaptic related genes families and (2) translation factors/activation. For details (i.e., methods, experimental design) on the studies included in this figure, refer to Tables 1, 2. To be included in this figure, a gene or protein had to show a similar trend in at least two independent studies. Genes in black represent mRNA expression. Genes highlighted in red means that expression (or activation) was also detected at the protein level.

**FIGURE 3:**
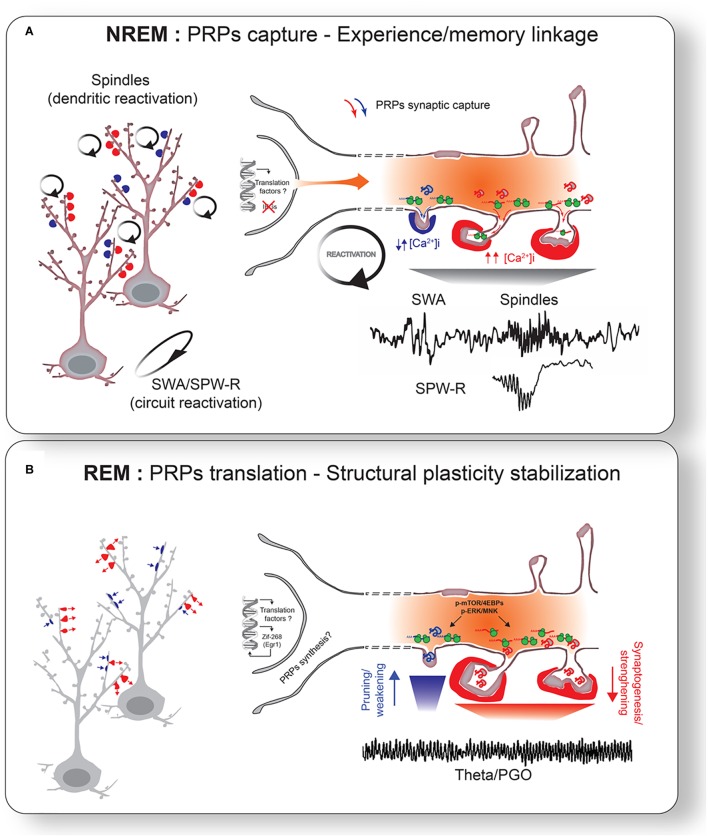
Sleep-stage specific consolidation mechanisms. **(A)** Oscillatory activity during NREM sleep triggers reactivation of primed neurons and synapses. Reactivation of tagged synapses promotes capture of PRPs. More localized spindle activity may in turn target reactivation of specific subset of dendrites. Different levels of intracellular Ca^2+^ may mediate PRPs capture for weakening or strengthening. This reactivation links neurons involved in different experiences. **(B)** Captured PRPs are translated into proteins to promote the final stage of structural plasticity stabilization. ERK and/or mTOR pathways may be particularly important for this process. This leads to bidirectional changes in synapses: strengthening of positively tagged synapses (red arrows) and weakening of negatively tagged synapses (blue arrows). REM sleep network activity (e.g., theta oscillations, PGO waves) likely participates for the stimulation of widespread translation activation. Transcription of translation factors and some IEGs (e.g., Egr1/Zif268) during NREM and REM sleep may also help sustained synaptic remodeling (i.e., structural plasticity) via replenishment of PRPs across NREM-REM cycles (Almeida-Filho et al., 2018).

The complete identity of PRPs is also unknown (Figure 2 and Table 1). An important feature of PRPs is their trafficking into dendrites (Cajigas et al., 2012) where they will be available near tagged synapses for subsequent capture upon reactivation (Figures 1B, 3A). The neurotrophin BDNF, the AMPAR subunit GluR1 and the kinase PKMzeta are the best characterized positive PRPs underlying persistent forms of LTP (Sajikumar et al., 2009; Sajikumar and Korte, 2011; Panja and Bramham, 2014). PSD-95, the increased expression of which at synapses is critical for persistent spine enlargement (Meyer et al., 2014), may also act as a positive PRP. Arc and Homer1a may act as tags, but have also been proposed to act as negative PRPs upon delayed network reactivation (Redondo and Morris, 2011). Arc and Homer1a are upregulated during wake in the cortex and hippocampus and have been linked to synaptic depression (Okada et al., 2009; Okuno et al., 2012; Jakkamsetti et al., 2013; Siddoway et al., 2014; Diering et al., 2017). The role of Arc in synaptic plasticity and memory is complex and manifold (Nikolaienko et al., 2018) but one important function at synapses is to promote internalization of AMPARs and LTD via interaction with other post-synaptic proteins such as endophilin/dynamin (Okuno et al., 2012; Wilkerson et al., 2018).

**Table 1:** Transcription and immediate early genes (IEGs) across sleep and wake in the cortex and hippocampus.

Reference	Methods	Manipulations	Genes	Wake/SD	Sleep
**Cortex**
Pompeiano et al., 1994; Cirelli et al., 1996	HIS, IHC	Spontaneous W and S (circadian)	c-fos, Egr1, p-CREB	↑ During periods enriched in wake	
Cirelli and Tononi, 2000a,b; Cirelli et al., 2004, 2006	ISH, RPA, IHC, Mi., qPCR, ELISA	Spontaneous W and S and SD	c-fos, FosB, JunB, Egr1/2/3/4, Nr4a1, CREB1, CREM, p-CREB, Arc, bdnf, Homer1a, narp, tPA	↑ In wake and SD	
Cirelli et al., 1995	HIS, IHC	SD	c-fos	↑ Compared to circadian controls	
Pompeiano et al., 1997	ISH	SD	Egr1		
Semba et al., 2001	IHC	SD	c-fos, JunB		
Nelson et al., 2004	qPCR	SD	Homer1a		
Mackiewicz et al., 2007	Mi.	SD	c-fos, Nr4a1, bdnf, Homer1a		
Huber et al., 2007b	qPCR	SD	Egr1, Arc, bdnf, Homer1a		
Delorme et al., 2018	qPCR, HIS, IHC	SD	Arc		
Taishi et al., 2001	qPCR, RPA	SD + 2 h S	Arc, bdnf, tPA	↑	↓
Terao et al., 2003	qPCR, IHC	SD + 4 h S	c-fos, Fra2, FosB, JunB, Egr1/3, Nr4a1	↑	↓ All ↑ Egr3
Hairston et al., 2004	Northern	SD + 2 h S	c-fos, bdnf	↑	↓
Terao et al., 2006	Mi., qPCR, northern	SD + 2 h S	c-fos, JunB, Egr1/3, Nr4a1, CREB1, Arc, bdnf, Homer1a, Narp MAP1B	↑	↓ All ↑ Egr3
Thompson et al., 2010	Mi., ISH	SD + 4 h S	c-fos, Egr1/3, Nr4a1/3, Arc, bdnf, Homer1	↑	↓
Del Cid-Pellitero et al., 2017	IHC	SD + 2 h S	Arc, c-fos	↑	↓
Ribeiro et al., 2007	ISH	EE + 2 h S or EE+ 4 h S or	Egr1, Arc	↑ With EE	↑ REM after EE
Ribeiro et al., 2002	ISH	Hippocampal LTP	Egr1	↑ With LTP	↑ REM after EE
Hanlon et al., 2009	IHC	Motor learning (ML) + 1 h S	c-fos, Arc	↑ With ML	↓ c-fos No change in Arc
Seibt et al., 2012	qPCR, WB	Visual experience (VE) + 1, 2, 6 h S	c-fos, Arc, bdnf		↓ mRNAs ↑ Arc and bdnf proteins
Calais et al., 2015	qPCR	EE + 1 h SD + 0.5 h S	c-fos, Nr4a1, Egr1, Arc	↑ With EE	↓
Genzel et al., 2017	qPCR	WM learning + 5 h S	c-fos, Egr1, Arc	↑ With WM	↓
Renouard et al., 2018	IHC	Visual experience (VE) + 1 h S/RSD	c-fos, p-CREB, Arc		↑ REM dependent c-fos, Arc = L2/3, L5/6 p-CREB = L4
**Hippocampus**
Pompeiano et al., 1994	HIS, IHC	Spontaneous W and S (circadian)	c-fos, Egr1	↑ During periods enriched in wake	
Luo et al., 2013	IHC	Spontaneous W and S (circadian)	p-CREB		↑ REM CA1 and DG
Cirelli et al., 1995	HIS, IHC	SD	c-fos	↑ Compared to circadian controls	
Vecsey et al., 2009	IHC	SD	p-CREB	↓ CA1 and DG	
Vecsey et al., 2012	Mi., qPCR	SD	c-fos, Nr4a1, CREB1, CREM, Arc	↑ Compared to circadian controls	↓ Arc
Delorme et al., 2018	qPCR, HIS, IHC	SD	Arc	↑ mRNA ↓ Protein (DG)	
Tudor et al., 2016	qPCR, WB	SD	Arc	↑ mRNA No protein change	
Hairston et al., 2004	Northern	SD + 2 h S	c-fos, bdnf	↑	↓
Thompson et al., 2010	ISH	SD + 4 h S	Nr4a1, Arc	↑	↓
Taishi et al., 2001	qPCR, RPA	SD + 2 h S	Arc	↑	↑
Ribeiro et al., 1999	ISH	EE + 2 h S	Egr1	No change	↑ REM after EE
Ribeiro et al., 2002	ISH	Hippocampal LTP	Egr1	↑ With LTP	No change
Ribeiro et al., 2007	ISH	EE + 4 h S	Egr1, Arc	↑ With EE	No change
Ulloor and Datta, 2005	WB	TWAA learning + 6 h S	p-CREB, Arc, bdnf	↑ During 6 h post TWWA, correlates with REM PGO	
Datta et al., 2008	qPCR, WB	TWAA learning + 3 h S (PGO inhibition)	Egr1, p-CREB, Arc, bdnf	↑ During 3 h post TWWA, dependent on REM PGO	
Calais et al., 2015	qPCR	EE + S	c-fos, Nr4a1, Egr1, Arc	↑ With EE	↑ REM after EE
Genzel et al., 2017	qPCR	WM learning + 5 h S	c-fos, Egr1, Arc	↑ With WM	↓


*Data reported in this table focus on changes in the cortex and hippocampus in mammals (rat, mouse, and cat). Changes in sleep–wake regulatory brain structures (e.g., hypothalamus, brainstem) and whole brain extract are not included. Waking data were restricted to periods of sleep deprivation <8 h. Underlined genes = protein (with or without mRNA; refer to the Methods column for details) were investigated. SD, sleep deprivation; S, sleep; RSD, REM sleep deprivation; EE, enriched environment; TWAA, two-way active avoidance; WM, water maze; Mi, microarray; qPCR, quantitative PCR; IHC, immunohistochemistry; HIS, *in situ* hybridization; RPA, Rnase protection assay; WB, Western blot; DG, dentate gyrus; Egr1, Zif268/NGF1-A; Nr4a1, Nur77/NGF1-B.*

Waking appears to be the preferred brain state for PRPs transcription and dendritic targeting of PRPs. This is suggested by two observations. First, many PRPs are synapse related IEGs (e.g., *arc*, *homer1a*, *bdnf, narp*) and thus rapidly transcribed in an experience-dependent manner. Second, a majority of genome-wide studies report that the transcription of these mRNAs is maximal during waking and drops during sleep (Figure 2 and Table 1). Therefore, in addition to the setting of tags, the waking brain state [and/or the circadian active phase (Noya, 2018)] may globally increase the pool of dendritic PRPs in neurons activated by experience (Figure 1).

#### Waking Changes in Intrinsic Excitability: Priming Neurons and Dendrites

Changes in neuronal excitability are achieved by modifying intrinsic membrane conductance in neuron cell bodies or specific neuronal compartments (i.e., dendrites) activated by experience (Hulme et al., 2013; Kastellakis et al., 2015; Lisman et al., 2018). Changes in excitability are often associated with synaptic plasticity (Armano et al., 2000; Daoudal and Debanne, 2003; Frick and Johnston, 2005; Xu et al., 2005) and can prime neurons by adjusting the threshold for subsequent plasticity. While several mechanisms can lead to increased excitability, an important contributor to this process is the cAMP/CREB pathway (Benito and Barco, 2010; Rogerson et al., 2014). Activation of CREB-dependent gene expression has been shown to lower the threshold for LTP expression and increase excitability via reduction of the afterhyperpolarization (AHP) current (Lopezde Armentia et al., 2007). This CREB-dependent increase in excitability is specific to neuronal circuits engaged in the learning task and enhances subsequent memory consolidation (Viosca et al., 2009; Zhou et al., 2009). cAMP also directly activates the hyperpolarization-activated cyclic nucleotide-gated (HCN) channels which are important regulators of excitability (Benarroch, 2013) and are expressed in high amount in distal dendrites of pyramidal neurons. This suggests that cAMP activation may result in greater excitability within dendrites (Losonczy et al., 2008) consistent with a role of HCN channels in localized dendritic plasticity (Williams et al., 2007; Shah, 2014). This is further supported by a recent study showing that transient plasticity induction in single distal dendrites leads to localized increases in membrane excitability in those dendrites (Sandler et al., 2016).

There are several findings suggesting that neuronal priming via increased excitability occurs during wakefulness. While the underlying physiology is not clear, cortical excitability in humans has been shown to increase with time spent awake (Huber et al., 2013; Meisel et al., 2015; Ly et al., 2016). This may also explain why sleep deprivation increases the risk for seizures (Gastaut and Tassinari, 1966; Meisel et al., 2015). At the cellular level, *in vivo* electrophysiological recordings in rodents have shown that both spontaneous neuronal firing (Vyazovskiy et al., 2009; Miyawaki and Diba, 2016) and evoked cortical response (Vyazovskiy et al., 2013) increase with waking time, consistent with increased neuronal excitability. Similarly, *in vitro* recordings in rodent cortical slices support a specific rise in mEPSCs frequency after prolonged wakefulness (Winters et al., 2011), paralleled by an increased firing probability in response to an injected current (Winters et al., 2011; Yan et al., 2011). This increase in neuronal excitability after extended wakefulness has also been linked to Ca^2+^-dependent AHP reduction (Yan et al., 2011). At the molecular level, CREB phosphorylation in the cortex, and associated IEG transcription occurs predominantly during wakefulness (Figure 2 and Table 1). It is therefore likely that in addition to enhancing PRPs availability, CREB activation during wakefulness modifies membrane excitability within circuits and dendrites activated by experience (Lisman et al., 2018) (Figures 1A,C).

## Bi-Directional Plasticity During Sleep: Capture and Translation of PRPs

### Bi-Directional Synaptic Plasticity and Sleep

*In vivo* electrophysiological recordings and dendritic spine imaging in rodents have provided evidence for bi-directional synaptic plasticity changes during sleep (reviewed in Frank and Cantera, 2014; Puentes-Mestril and Aton, 2017; Navarro-Lobato and Genzel, 2018). Recordings of neuronal spiking, an indirect measure of plasticity, shows both increased and decreased firing rates in the hippocampus and cortex during sleep. More specifically, while NREM sleep is accompanied by more heterogeneous and bi-directional changes in spiking rates (Chauvette et al., 2012; Grosmark et al., 2012; Miyawaki and Diba, 2016; Watson et al., 2016; Norimoto et al., 2018), firing rates tend to decrease across individual REM sleep episodes (Grosmark et al., 2012; Miyawaki and Diba, 2016; Watson et al., 2016). However, increased firing rate following exposure to novel perceptual experience has been shown to occur during both sleep stages in sensory cortical areas (Ribeiro et al., 2007; Aton et al., 2014; Clawson et al., 2018).

When measured *after* sleep, circuits can be strengthened or weakened (reviewed in Frank and Cantera, 2014). For example, sleep-dependent changes in ODP induced by monocular deprivation (as measured by evoked electrophysiological responses) involve a gain of response to the open eye and a weakening of response to the deprived eye (Aton et al., 2009a; Seibt et al., 2012). Although decreases in evoked electrophysiological responses to stimuli are reported after sleep in some rodent studies (Vyazovskiy et al., 2008), this is not always found. For example, in the cat, electrically evoked cortical responses measured during wake are larger after a period of sleep (Chauvette et al., 2012). Electrophysiological recordings also show that sleep is required for an experience-dependent form of LTP (Cooke and Bear, 2010, 2012) in the adult visual cortex *in vivo* (Aton et al., 2014; Durkin and Aton, 2016). Some of the heterogeneity in firing rates after sleep may also be explained by the class of neurons recorded (i.e., inhibitory vs. excitatory) and the basal level of firing rate (i.e., low vs. high) (Levenstein et al., 2017; Clawson et al., 2018).

Recent *in vivo* imaging studies, which provide more direct measurements of synaptic plasticity, also show complex changes in spine structure during sleep and suggest a role for sleep in stabilization of both spinogenesis and spine pruning (reviewed in Sigl-Glöckner and Seibt, 2018). Sleep during adolescence, when spine turn-over and pruning are high, seems to favor spine pruning in some cortical areas (Maret et al., 2011; Yang and Gan, 2012). Spine pruning during development is central to the refinement of synaptic connections in response to experience (Zuo et al., 2005; Yu et al., 2013). During adulthood, when spines are much more stable, periods of sleep following experience have been shown to be important for both synaptic strengthening/spinogenesis and synaptic weakening/spine pruning (Yang et al., 2014; Li et al., 2017), consistent with long-term structural modifications at synapses. This effect seems to mainly affect recently formed spines, leaving more stable ones relatively unaffected by sleep (Yang et al., 2014; Li et al., 2017). Furthermore, data in the developing rodents further suggest that sleep may favor the pruning of smaller spines in the cortex (Vivo et al., 2017). A similar trend has been recently described *in vitro* in the hippocampus in immature mice (Norimoto et al., 2018). While speculative, it is possible that the large and small spines represent the positive and negative tagging status of synapses, respectively. In the context of our model, this would further suggest that sleep might be particularly important for the final stabilization of these bi-directional transient changes in spine morphology induced during plasticity induction.

Specific cellular changes during NREM sleep may also be predictive of plastic change during REM sleep. For example, increased power in specific NREM oscillations associated with plasticity (i.e., spindles) correlate positively with changes in Ca^2+^ activity in cortical dendrites (Seibt et al., 2017) and CaMKII phosphorylation in hippocampal neurons (Blanco et al., 2015) during REM sleep. These results suggest that experience may affect neurons and circuits differently during both sleep stages but that these changes are most likely complementary. This is also in agreement with existing two-stage models emphasizing an important role for NREM-REM sleep in memory consolidation (see the section “Comparisons and Contrasts With Other Theories” for discussion). In the following sections, we discuss how different sleep stages may cooperate for the capture and translation of PRPs on a background of cell-specific increase in intrinsic excitability (Figure 3).

### NREM Oscillations for PRPs Capture (Figure 3A)

According to current ideas about the STC, the first and necessary step toward stabilization of functional and structural plasticity is the capture of PRPs during the lifetime of the tag (minutes to hours) (Redondo and Morris, 2011). We propose that periodic reactivation of task-specific circuits during NREM oscillations, combined with priming during wake, provides an efficient mechanism for PRPs capture in a neuron- and synapse-specific manner. Localized changes in excitability can determine which dendritic segment or group of synapses are more likely to engage in synchronization patterns within a given circuit (Alarcon et al., 2006). Since reactivation affects both positively and negatively tagged synapses, this would also explain how sleep leads to bi-directional changes in synaptic plasticity. In our view, what matters is the presence of these oscillations, rather than their form which reflects differences in the underlying circuitry. NREM oscillations *in general* can promote synaptic capture of PRPs because they all involve a reactivation of a network and they increase in number or intensity in a use-dependent fashion.

This idea is supported by several observations. Three main NREM sleep oscillations have been implicated in experience-dependent plasticity and memory consolidation: slow-wave activity (SWA; 0.5–4 Hz) and spindles (sigma: 10–16 Hz) in the cortex, and sharp wave ripples (SPW-R; 100–200 Hz) in the hippocampus (Rasch and Born, 2013; Buzsáki, 2015; Ulrich, 2016). Although these oscillations are generated by different neuronal populations within the thalamocortical (i.e., spindles and SWA) and hippocampal (i.e., SPW-R) circuits, they all share common physiological and functional features. SWA, spindles and SPW-R have been shown to be temporally coupled across brain regions (Siapas and Wilson, 1998) and are all increased (either in number or intensity) following learning and experience (Rasch and Born, 2013; Ulrich, 2016; Norimoto et al., 2018). These use-dependent changes in NREM oscillations have been clearly demonstrated for cortical SWA and spindles in humans and animals (Vyazovskiy et al., 2000; Huber et al., 2004, 2006, 2007a; Eschenko et al., 2006; Mölle et al., 2009; Johnson et al., 2012; Cox et al., 2014). Some of these oscillations have also been shown to include coordinated reactivation of neurons and synapses previously engaged in learning tasks (Bergmann et al., 2012; Chen et al., 2013; Sadowski et al., 2016). The best evidence of this reactivation (or “replay”) occurs in hippocampal cells during SPW-R (Buzsáki, 2015), but NREM replay is also reported in cortical ensembles (Euston et al., 2007; Ji and Wilson, 2007).

Waking experience has also been shown to increase synchronization and coherence in the activity of task-related neurons in the hippocampus and cortex during sleep (Gulati et al., 2014; Durkin et al., 2017; Ognjanovski et al., 2017). This increase in NREM network synchronization also correlates with plasticity measures and memory performance (Gulati et al., 2014; Ognjanovski et al., 2018). There is also evidence that the latter events are linked to neuronal priming and necessary for PRPs capture. Constitutive CREB activation (which increases excitability), via CAMKIV overexpression in mice increases NREM SWA (Steenland et al., 2010).

The direction of plastic change during sleep is likely influenced by the neurons’ recent waking activity history, which in turn determines the setting of different tags. For example, SPW-R during sleep appears to induce synaptic weakening *in vivo* only in neurons that were *not* engaged in a previous novel experience (Norimoto et al., 2018). Similarly, only neurons that are highly responsive during a motor task will show increased SWA spiking coherence during subsequent sleep (Gulati et al., 2014). In addition, while spindle activity has been linked to increased plasticity *in vitro* (Rosanova and Ulrich, 2005) and *in vivo* (Timofeev et al., 2002), more recent data also show that spindles predict global decreases in firing rate across sleep stages (Miyawaki and Diba, 2016). Consistent with a previous hypothesis (Contreras et al., 1997; Steriade and Timofeev, 2003), Seibt et al. (2017) recently demonstrated using *in vivo* Ca^2+^ imaging that spindles might be particularly important for synchronized reactivation of dendritic populations.

Given its important role as a sensor of synaptic activation, different levels of intracellular Ca^2+^ in different groups of synapses during NREM oscillations may mediate PRPs capture weakening or strengthening. Using two-photon imaging in anesthetized mice, Chen et al. (2013) demonstrated that spines that displayed increased Ca^2+^ during UP states of slow oscillations represented the same population that showed Ca^2+^ increased during previous sensory evoked responses. Although performed in anesthetized mice, this supports the idea that synaptic tags are set during waking and captured during subsequent NREM sleep. Consistent with bi-directional plasticity changes during NREM sleep, it was recently shown that individual cortical dendrites display both increased and decreased Ca^2+^ activity during spindles (Seibt et al., 2017). This further suggests that local changes in Ca^2+^ levels during spindles may trigger intracellular mechanisms that favor both potentiation and depression in a dendrite-specific manner.

### REM Sleep Translation of PRPs Stabilizes Structural Plasticity

Recent studies using two-photon imaging of single dendritic spines in mice provide direct evidence that sleep stabilizes experience-dependent structural plasticity during development and adulthood (Yang et al., 2014; Li et al., 2017). In both studies, lack of sleep disrupted both motor learning and structural plasticity stabilization. As mentioned before, an important step for the stabilization of labile into more permanent synaptic changes is new protein synthesis. More specifically, localized mRNA translation near/at synapses is a key mechanism neurons use to promote synapse-specific structural modifications during development and adulthood (Holt and Schuman, 2013). Our model proposes that, while experience-dependent PRPs transcription occurs predominantly during wake, the final translation of PRPs transcripts necessary for synapse-specific structural plasticity stabilization occurs during REM sleep (Figure 3B).

Translation activation in the brain is specifically enhanced during sleep (Table 2) and is accompanied by increased expression of PRPs proteins (Figure 2). Among the multiple pathways involved in translation regulation for plasticity and memory, one important step is activation of the eukaryote initiation factor 4E (eIF4E) resulting in translation initiation activation (Richter and Sonenberg, 2005). Two main pathways can lead to eIF4E activation: the mammalian target of rapamycin (mTOR)/4E-BPs and ERK/Mnk1 pathways (Banko and Klann, 2008). In addition to showing increased cortical and hippocampal activation during periods dominated by sleep (Eckel-Mahan et al., 2008; Seibt et al., 2012; Vecsey et al., 2012; Dumoulin et al., 2015; Luo et al., 2013; Tudor et al., 2016; Diering et al., 2017), inhibition of these pathways in the visual cortex of cats during sleep impairs the consolidation of plastic changes during ODP (Seibt et al., 2012; Dumoulin et al., 2015). A similar inhibition during waking had no effect on the labile changes triggered by monocular deprivation (Seibt et al., 2012; Dumoulin et al., 2015). Furthermore, inhibition of mTOR signaling during sleep prevents the enhancement of synaptic potentiation *and* reverses the depression in visual circuits normally seen during ODP (Seibt et al., 2012). This is consistent with a role for translation in stabilization of bi-directional structural plastic changes. Finally, memory impairment induced by sleep deprivation can be rescued by enhancing 4E-BP2 activity in the hippocampus of sleep deprived mice (Tudor et al., 2016).

**Table 2:** Translational activity in the cortex and hippocampus across sleep and wake.

Reference	Methods	Area	Manipulations	Measures	Wake/SD	Sleep
Cirelli et al., 2004	Mi.	Cortex	Spontaneous W and S and SD	eEF2, eIF4AIII		↑
Mackiewicz et al., 2007	Mi.	Cortex	SD	eEF2, eIF4b, eIF5, eIF3		↑
Naidoo et al., 2005	Ribosome profiling	Cortex	SD	General translation	↓ Polysomes	
Naidoo et al., 2008	WB	Cortex	SD	p-eIF 2	↑ (↓ Translation activity)	
Seibt et al., 2012	WB	Cortex	Visual experience + 1, 2, 6 h S	p-4EBP1, p-eEF2		↑ @ 1–2 h sleep
Renouard et al., 2018	IHC	Cortex	Visual experience + S and RSD	p-mTOR		↑ REM dependent
Vecsey et al., 2012	Mi., qPCR	Hipp.	SD	eIF2a, eIF3s6ip, eIF4e2, eIF5, Rbm3, Denr	↓	
Vecsey et al., 2012	WB	Hipp.	SD + 2.5 h S	p-mTOR	↓	↑
Tudor et al., 2016	WB	Hipp.	SD	p-mTORC 1, p-4EBP2, eIF4E/eIF4G	↓	
Tudor et al., 2016	WB	Hipp.	SD	Puromycin (SUnSET)	↓	


*Data reported in this table focus on changes in the cortex and hippocampus in mammals (rat, mouse, and cat). Changes in sleep–wake regulatory brain structures (e.g., hypothalamus, brainstem) and whole brain extract are not included. Waking data were restricted to periods of sleep deprivation <8 h. Hipp., hippocampus; SD, sleep deprivation; S, sleep; RSD, REM sleep deprivation; Mi, microarray; qPCR, quantitative PCR; IHC, immunohistochemistry; WB, Western blot.*

These results in mice and cats suggest that the increase in translation initiation during sleep is particularly critical for brain plasticity and memory consolidation. But are PRPs actually translated during sleep? In contrast to their transcripts, protein expression of several PRPs increase or remain at high levels during sleep in the hippocampus and cortex [i.e., BDNF, Arc, PSD95, Glur1; Figure 2 and (Ulloor and Datta, 2005; Seibt et al., 2012; Dumoulin et al., 2015; Del Cid-Pellitero et al., 2017; Renouard et al., 2018)]. Inhibition of mTOR and ERK signaling during sleep also decreases PRPs protein levels (Seibt et al., 2012; Dumoulin et al., 2015) suggesting a regulation of expression at the translational level. Activation of translation initiation and increased protein expression of some PRPs during sleep are also more pronounced at synapses supporting a synaptic-specific mechanism (Seibt et al., 2012).

REM sleep appears to be a preferred time for PRPs translation and structural plasticity. In mice, sleep-dependent structural plasticity is promoted by REM sleep during development and after learning (Li et al., 2017). Enzymes regulating translation and expression of PRPs proteins during sleep seem to also depend specifically on REM sleep. In the mouse hippocampus, the increase in ERK phosphorylation during the light (rest) phase (Eckel-Mahan et al., 2008) is maximal after a period rich in REM sleep (Luo et al., 2013). REM sleep deprivation in cats significantly decreases cortical ERK phosphorylation (Bridi et al., 2015). The cellular mechanisms by which REM sleep promotes translation are not known but may be supported by the specific network activity occurring during that stage. In the hippocampus, increased Arc and BDNF proteins after learning correlates with amounts of REM sleep pontine-geniculate-occipital waves (PGO) (Ulloor and Datta, 2005) (Table 1). Protein synthesis in dendrites of hippocampal CA1 neurons can be elicited *in vitro* with electrical stimulation in the theta (4–10 Hz) range (Feig and Lipton, 1993). Interestingly, dendritic protein synthesis in this model was dependent on pharmacological activation of muscarinic receptors, suggesting a role for the high cholinergic tone during REM sleep in translation activation at synapses. This observations are consistent with recent *in vivo* data showing an important role for REM sleep theta oscillations for memory consolidation (Boyce et al., 2016; Ognjanovski et al., 2017). Furthermore, both synaptic potentiation and depression can be elicited depending on the theta oscillatory phase (i.e., peak vs. through) at which electrical stimulation is applied (reviewed in Poe et al., 2010).

## Implications of the Model

### Selection and Association of Memories During Sleep

Immediate activation of consolidation upon learning is not optimal at the cellular and system levels. As discussed in Redondo and Morris (2011), consolidation of successive experiences at the time of encoding would result in superimposed activation of multiple cellular consolidation cascades within neurons. This would require extremely tight temporal regulation and high energy demands. This would also prevent selection of salient information and experience as all incoming stimuli would be stored (creating interference and a signal-to-noise problem). This problem, albeit expressed in different ways, was recognized many decades ago by scientists interested in the role of sleep in memory consolidation (reviewed in Alger et al., 2015). Sleep theoretically solves the problem as incoming stimuli are dampened and an internal reactivation of primed circuits ensures a very high signal-to-noise ratio during the consolidation process.

An advantage of the STC model is that cellular consolidation mechanisms are triggered within successive check points in time by binding significant experiences within shared circuits and neurons using shared cellular pathways. We propose in our model that these check points occur in sleep, when the brain is partially disengaged from incoming information and experience. This is consistent with the idea that memories are not just consolidated during sleep but are also actively reorganized to help cognitive processes such as generalization (Stickgold and Walker, 2013) or creative thinking and problem solving (Lewis et al., 2018). The ways by which sleep may achieve this is not clear but mechanisms of selective “memory triage” (Stickgold and Walker, 2013) and network association (Cai et al., 2009) have been proposed. Linked to this idea, part of the memory consolidation process involves the integration of new experience into pre-existing memory engrams (Rasch and Born, 2013) which would require the sharing and association of common circuits. While some theories have implicated REM sleep for memory association (Lewis et al., 2018), we propose that this process may particularly benefit from PRPs capture during NREM sleep (Figure 3A). The STC model and CREB-dependent increases in neuronal excitability have been proposed to facilitate associative memory (i.e., memory allocation) through the activation of shared circuits and synapses by experiences close in time (Govindarajan et al., 2006; Silva et al., 2009; Redondo and Morris, 2011; Rogerson et al., 2014; Lisman et al., 2018). If two experiences activate partially overlapping circuits, this leads to the tagging of synapses close in space (e.g., clustered on the same dendrites) and the co-activation of some neurons shared by these experiences (Figure 1A). During subsequent NREM sleep reactivation, different experiences can then be linked throughout the network. Enhanced CREB-dependent excitability will bias these neurons to be reactivated together during sleep and favor their linkage (Lisman et al., 2018). Similarly, synapses activated at close proximity will share the same type of tags and PRPs and will more likely strengthen (or weaken) in clusters, an important mechanism of memory allocation and storage (Rogerson et al., 2014; Kastellakis et al., 2015).

### Linking Synaptic Plasticity and Sleep Homeostasis

Our model provides a new way to connect waking experience to sleep homeostasis. As discussed by Benington, any proposed function of sleep should describe how the progress of that function determines sleep need (Benington, 2000). In our model, neurons primed during waking experience will express more (or more intense) modulation by NREM sleep oscillations. Over the course of the sleep period, this leads to the capture, translation, and removal of the priming elements, which also reduces the electrophysiological metrics of sleep homeostasis. Thus, in contrast to other hypotheses linking, for example, NREM SWA to plasticity (Tononi and Cirelli, 2003, 2006), heightened SWA is not caused by stronger synapses in wakefulness, nor does it result only in synaptic weakening during sleep. SWA increases are instead caused by the priming of neurons differentially engaged during waking experience. As this priming includes positive *and* negative tagging, subsequent increases in NREM SWA lead to synaptic weakening and strengthening, depending on the synaptic tag. If this is true, one would predict that changes in priming (i.e., excitability and tags) parallel changes in sleep need. There is evidence to support this idea. Increased synaptic phosphorylation, which is involved in synaptic tagging according to our model (see the section “Tags”), parallels increases in sleep need and decreases during sleep (Wang et al., 2018). PRPs transcript levels generally are maximal during waking and then decline during sleep (Figure 2 and Table 1). Several IEGs show a peak and decline in their cortical expression following SD and recovery sleep that parallels the time constant for the discharge of sleep homeostasis (as measured by SWA) (Gerstner et al., 2016). In addition, some of the more dramatic sleep phenotypes in mutant animals result from changes in membrane excitability [e.g., potassium channels in drosophila (Cirelli et al., 2005; Bushey et al., 2007; Koh et al., 2008; Wu et al., 2014) and mice (Espinosa et al., 2004; Douglas et al., 2007)].

### Comparisons and Contrasts With Other Theories

The goal of this article is not to exhaustively review all other theories about sleep and plasticity. However, it is useful to put our theoretical model in context. Other theorists have proposed roles for REM and NREM sleep in synaptic plasticity and memory consolidation. For example, at a systems level, it has been proposed that NREM sleep oscillations transfer and propagate memory traces from the hippocampus to neocortical areas (Ribeiro and Nicolelis, 2004; Rasch and Born, 2013; Navarro-Lobato and Genzel, 2018). REM sleep episodes could then promote consolidation of the transferred engram at the molecular level, mainly via transcriptional events (Ribeiro and Nicolelis, 2004; Rasch and Born, 2013; Grønli et al., 2014; Navarro-Lobato and Genzel, 2018). Some models also include a role for translation during sleep (Grønli et al., 2014; Navarro-Lobato and Genzel, 2018). However, unlike our model, this is primarily linked to NREM sleep and promotes global restorative biogenesis and local synaptic strengthening (Grønli et al., 2014).

Our model is broadly compatible with these ideas, but distinct in important ways. We propose that the bulk of transcriptional events occur during waking and is necessary for PRP synthesis at the transcriptional, not translational level. We focus on synaptic changes that may accompany systems level changes, thus our ideas are not incompatible with the transfer of information from the hippocampus to the cortex. We add to the proposed function of NREM sleep oscillations in that we suggest PRP capture at selected synapses in reactivated circuits (regardless of brain structures). In contrast to other ideas positing different roles for REM and NREM sleep in plasticity (Crick and Mitchison, 1983; Giuditta et al., 1995; Rasch and Born, 2013; Giuditta, 2014; Almeida-Filho et al., 2018; Navarro-Lobato and Genzel, 2018), our ideas are based on current ideas about metaplasticity, rather than uniform processes that drive plasticity in one (net) direction, or transcriptional events only. Our model also differs in the description of priming mechanisms that link plastic changes in waking with subsequent changes in sleep.

Our model also differs from the ‘Synaptic Homeostasis Hypothesis (SHY)’ which proposes that sleep weakens synapses while sparing others (a process referred to as ‘down-selection’), resulting in a ‘net’ weakening of synapses after sleep. This type of plasticity has also been termed ‘downscaling’ (Tononi and Cirelli, 2003) and described in terms similar to homeostatic plasticity (Turrigiano, 2008). Although SHY allows for some synapses to become stronger during sleep, this occurs under special (e.g., non-physiological) conditions, and therefore is not a major aspect of SHY (Tononi and Cirelli, 2014). Finally, according to SHY ‘protection’ from down-selection does not involve an absolute increase in synaptic strength; merely a relative change compared to the net downscaling of other synapses in sleep (Tononi and Cirelli, 2014; Cirelli and Tononi, 2015). Our model instead states that synaptic strengthening is just as likely to occur as weakening during sleep (depending on prior waking experience), is not necessarily relative to other synapses, does not require ‘net’ synaptic downscaling and reflects physiological and adaptive processes.

This does not make our model incompatible with synaptic homeostatic adjustments during sleep. Some evidence cited in support of SHY may be explained by circadian rhythms rather than sleep (Frank and Cantera, 2014). Other findings suggest that some forms of homeostatic plasticity may only occur during wakefulness (Hengen et al., 2016). Some synaptic changes ascribed to ‘net’ downscaling may instead reflect the influence of negative tags and PRPs as we describe. Nevertheless, homeostatic adjustments of synapses during sleep may occur but in a manner that more generally restores set-points of activity and synaptic weight upward or down-ward depending on the neuron (Watson et al., 2016; Levenstein et al., 2017). In addition, as shown recently in a study of inhibitory and excitatory receptors on the soma of cortical principal neurons (Del Cid-Pellitero et al., 2017), homeostatic scaling in sleep could provide a global means of adjusting excitability. In this context, this might reflect a sleep-dependent and bi-directional restorative process, reflecting the neurons’ previous waking history. This homeostatic process would not interfere with Hebbian synaptic plasticity on individual synapses, neurons or circuits, as originally proposed (Turrigiano, 2008).

## Looking Into the Future: Open Questions and Future Directions

According to our model, labile forms of plasticity are induced when we are awake and are consolidated into more permanent forms while we sleep. Waking plasticity involves transient plastic changes and priming mechanisms (changes in excitability and synaptic tagging). Waking plasticity also includes transcription and dendritic targeting of PRPs (i.e., IEGs) for synaptic capture during sleep. Plasticity during sleep promotes capture and translation of PRPs at synapses for stabilization of structural plasticity. Our model, informed by the STC model and new discoveries regarding priming, provides a molecular bridge between plastic changes induced in one state (wakefulness) and the other (sleep). Although speculative, this model explains a number of disparate findings in the field and leads to falsifiable predictions.

### Experimental Considerations, Predictions, and Unanswered Questions

#### Experimental Considerations

In this article, we draw on many sources of data, ranging from *in vitro* to *in vivo* experiments, and studies in many different animal species often in different brain regions. It is important to consider some general caveats in how one interprets these data. Many experiments use SD as a model of the natural waking brain state. SD, however, can increase stress levels and stress hormones can influence metaplasticity (Schmidt et al., 2013), transcription and translation regulation (Finsterwald and Alberini, 2014; Grønli et al., 2014) as well as structural plasticity (Spruston, 2008; Liston and Gan, 2011). Furthermore, while waking plasticity may be linked to sleep as we and others (Tononi and Cirelli, 2014; Wang et al., 2018) propose, SD may affect neuronal function in a different and more global manner leading to activation of pathways unrelated to plasticity (Naidoo, 2012; Vyazovskiy and Harris, 2013). It will therefore be critical in future experiments to minimize and control for side effects due to enforced wakefulness.

Another important consideration is the interaction of circadian rhythms with the sleep–wake cycle. Biological clocks can influence sleep, transcription and translation regulation, as well as functional and structural plasticity (Frank and Cantera, 2014). For example, diurnal oscillations in ERK phosphorylation and corticosterone levels directly influence learning capacity (Eckel-Mahan et al., 2008) and structural plasticity (Liston and Gan, 2011; Liston et al., 2013), respectively. Circadian rhythmicity has also been shown to contribute to changes in levels of neuronal excitability (discussed in Frank and Cantera, 2014; Frank, 2016). It will thus be critical to clarify the respective role of biological rhythms (i.e., sleep–wake and circadian cycles) and/or their synergistic effect, on any cellular and molecular mechanisms investigated.

Some of the strongest evidence of sleep’s role in brain plasticity comes from developing animals. It is well established that plasticity in the developing brain differs not only in degree, but kind, from plasticity in the adult brain. One example is plasticity during critical periods, when intracortical inhibition plays a much larger role than it does in the adult brain (Frenkel and Bear, 2004; Espinosa and Stryker, 2012). Compared to adulthood, the developing brain also exhibits higher spine turnover accompanied by an overall programs of enhanced synaptogenesis followed by synaptic pruning (Bhatt et al., 2009). The amounts, brain activity and homeostatic regulation of sleep also undergo dramatic changes in development (Davis et al., 1999). REM sleep, for example, is maximal at ages when waking experience is minuscule and may even be present without NREM sleep (Davis et al., 1999). This challenges predictions and assumptions of any model of sleep-dependent plasticity, including our own. Therefore, it is possible that not all findings obtained from developing animals will generalize across the lifespan. Finally, a major problem in any *in vivo* research is the methodology used to measure the physiological read-out. In the case of synaptic plasticity, molecular and electrophysiological techniques are by far the leading approaches. However, many of these measures are indirect [e.g., firing rate (Frank and Cantera, 2014)] and the results can be interpreted in different ways. This is because many of the molecular markers of plasticity are involved in different cellular functions and shared among various intracellular pathways. A notable example is Arc, a key regulator of long-term plasticity and memory implicated in LTP, LTD, synaptic homeostasis, and regulation of transcription (Minatohara et al., 2016; Nikolaienko et al., 2018). Therefore, changes in Arc observed after sleep could reflect several different processes. Given the increasing evidence that sleep is involved in subtle and synapse-specific regulation of plasticity, future experiments may greatly benefit from advances in *in vivo* imaging techniques that, combined with genetic tools and electrophysiological approaches, will allow scientists to address this issue in a more direct manner (Sigl-Glöckner and Seibt, 2018).

#### Predictions

Our model proposes that wake and sleep support different, but complementary, mechanisms which together lead to the full expression of long-term synaptic plasticity. This leads to several predictions:

• If neuronal priming is related to sleep need, then it should be proportional to time spent awake and should reverse as sleep need discharges. This prediction is supported by some evidence, but this should be investigated in more detail using appropriate methods for cellular (i.e., intrinsic excitability measures) and molecular (i.e., tag and PRPs) correlates of priming.
• If NREM oscillations are necessary for the synaptic capture of PRPs, manipulating these oscillations should alter the synaptic mRNA content. In other words, NREM oscillation disruption should result in more PRPs in the cytoplasm (soma and dendrites) compared to synapses. Conversely, enhancing these oscillations should increase synaptic PRPs content while decreasing their presence in the cytoplasm.
• If REM sleep increases synaptic mRNA translation, then manipulating REM sleep amounts should modify the synaptic proteomic content. In addition, given the high amount of REM sleep during early life (Jouvet-Mounier et al., 1970), it is expected that sleep-dependent mRNA translation is greatly enhanced in early life.

#### Unanswered Questions

Our model resolves some contradictions in the field and provides a novel way of thinking about how plasticity is divided across brain states. Nevertheless, working scientific models can oversimplify complex systems and should be cautiously interpreted. There are several findings that currently do not fit with the proposed model. While it is empirically useful to divide the synthesis, capture and translation of PRPs to wake, NREM and REM sleep, it is not clear that this division of labor always occurs. Transcription of certain IEGs (e.g., Arc, Egr1, and Egr3) also increase during REM sleep in the hippocampus and cortex (Figure 2 and Table 1). For example, the transcription factor Egr1 (i.e., Zif268) has been shown to increase during both wake *and* REM sleep following exposure to an enriched experience (Ribeiro et al., 1999, 2007). Zif268 is known to influence memory and LTP consolidation (Veyrac et al., 2014) and has been shown to bind to numerous targets, many of them involved in synaptic reorganization and stabilization (e.g., Arc, PSD-95, synapsin 1–3, translation factors; reviewed in Duclot and Kabbaj, 2017). One interpretation of this reactivation of PRPs synthesis is that REM sleep not only leads to consolidation in some circuits, but may also promote a re-opening of a labile, plastic period, in a manner like reconsolidation in hippocampal based memory. This secondary induction phase may also replenish primed neurons with PRPs for capture during sequences of NREM-REM sleep cycles. This might explain why REM sleep is more abundant at later stages of the sleep cycles, when the available pool of PRPs synthesized during waking may be reaching a nadir.

It is also not clear if all or most synaptic translation occurs during REM sleep. Studies using the uptake of radio-labeled amino-acids show positive correlations with NREM sleep and cortical protein synthesis (Ramm and Smith, 1990; Nakanishi et al., 1997; Czikk et al., 2003). It is possible that NREM and REM sleep both promote protein synthesis serving different functions such as macromolecule biosynthesis for cellular restorative function (Mackiewicz et al., 2007; Vyazovskiy and Harris, 2013) and structural plasticity at synapses, respectively. It is also possible that synapse-specific translation of different pools of mRNAs occurs during both the encoding (wake) and consolidation (sleep) phases of plasticity. Rapid translation near spines increase transiently (<2 h) after *in vivo* hippocampal LTP induction (Ostroff et al., 2018) and has been hypothesized to be also part of the tagging mechanism (Redondo and Morris, 2011). Furthermore, learning and LTP protocols induce several waves of protein synthesis *in vivo* (reviewed in Frey and Frey, 2008). Interestingly, it seems that these waves depend on different translation regulation pathways, with the later waves (>3 h) favoring dendritic translation controlled by the 4EBPs/eIF4E complex (Panja and Bramham, 2014). This suggests that early and late plasticity may require translation that depends on proteins with different functions. Future experiments using cell-specific and compartment-specific measures of the translatome and/or proteome will be necessary to clarify the role of translation in wake and sleep stage-dependent plasticity.

## Author Contributions

JS wrote the manuscript and prepared the figures. MF helped to write the manuscript.

## Conflict of Interest Statement

The authors declare that the research was conducted in the absence of any commercial or financial relationships that could be construed as a potential conflict of interest.
